# Clinical and Sociodemographic Profile of Patients Attending a Public Assisted Reproduction Unit and Factors Associated with the Type of Technique Used

**DOI:** 10.3390/healthcare14162653

**Published:** 2026-08-21

**Authors:** Lucía Inmaculada Martín Román, Ana Cerezo Mondragón, Ana Gallardo Carvajal, Isaac Cohen Corcia, Marta Blasco Alonso, Jesus S. Jimenez Lopez

**Affiliations:** 1 Department of Surgical Specialties, Biochemistry and Immunology, University of Malaga, 29010 Malaga, Spain; luciamartin424@gmail.com; 2 Obstetrics and Gynecology Department, Hospital Materno-Infantil, Hospital Regional Universitario Malaga, Avenida Arroyo de los Angeles S/N, 29011 Malaga, Spain; annecerezo@hotmail.com (A.C.M.); ana_gc_85@hotmail.com (A.G.C.); icohenc@gmail.com (I.C.C.); 3 Research Group in Maternal-Foetal Medicine Epigenetics Women’s Diseases and Reproductive Health, Biomedical Research Institute of Malaga (IBIMA), 29010 Malaga, Spain

**Keywords:** infertility, assisted reproductive techniques, sociodemographic profile, uterine factor, public health system

## Abstract

Objective: To describe the clinical and sociodemographic profile of patients treated in a public assisted reproduction unit and to analyze the association between these characteristics and the type of assisted reproductive technique used. Materials and Methods: A retrospective, observational, descriptive-analytical study was conducted including patients who initiated or underwent assisted reproductive techniques at a tertiary public hospital in Andalusia between January and June 2025. Sociodemographic and clinical variables were collected from electronic medical records and analyzed using bivariate and multivariate models. The main outcome was the type of assisted reproductive technique used. Results: A total of 222 patients were included. The mean age was 34.70 years (SD 3.99), and most were employed, nonsmokers, and of normal weight. Heterosexual couples were the most frequent family model, although single mothers by choice and female couples also represented a substantial proportion of the cohort. In the adjusted multivariable model, older maternal age (aOR = 1.19, *p* = 0.004), male factor infertility (aOR = 5.07, *p* = 0.026), and mixed factor infertility (aOR = 5.43, *p* = 0.017) were significantly associated with higher odds of undergoing high-complexity techniques. Conversely, urban residence (aOR = 0.24, *p* = 0.003) and single motherhood by choice (aOR = 0.12, *p* = 0.010) were significantly associated with lower odds of high-complexity techniques (and higher reliance on low-complexity procedures). Educational level, BMI, smoking status, and structural uterine pathology showed no independent association with technique complexity. Structural uterine pathology was among the most frequent gynecological findings, but no independent association with high-complexity techniques was demonstrated in the adjusted analysis. New family models accounted for 23.6% of the cohort, including 16.67% single mothers by choice and 6.94% female couples. Conclusions: In this cohort, the type of assisted reproductive technique used was mainly associated with clinical factors and family model rather than with the sociodemographic variables analyzed. These findings describe patterns of technique selection in a public assisted reproduction unit, but do not directly assess access to care.

## 1. Introduction

In public assisted reproduction systems, the type of technique utilized not only reflects clinical complexity but also illustrates how the health system manages access and prioritizes care. Numerous studies have demonstrated that age, educational attainment, family structure, geographic residence, and other social determinants can influence both the likelihood of accessing treatment and the specific technique ultimately prescribed. In this context, evaluating the profile of patients managed in a public unit may help describe treatment patterns within the public system, but it does not by itself allow conclusions about equity in access. In Andalusia, public reproductive care operates within the framework of the Andalusian Health Service (Servicio Andaluz de Salud), providing public funding and clinical indication criteria designed to prioritize medical need, although potential organizational and social barriers persist that warrant empirical investigation [1,2].

Infertility is defined as a disease of the reproductive system characterized by the failure to achieve a clinical pregnancy after twelve or more months of regular, unprotected sexual intercourse. The most recent international terminology expands this concept to include the necessity for medical intervention to achieve pregnancy in individuals or couples whose reproductive circumstances preclude conception without assistance, including specific family models requiring donor gametes [3].

Over recent decades, the prevalence of infertility has steadily increased in Western societies, currently affecting approximately 15% of couples of reproductive age. This condition represents not only a medical challenge but also a significant psychological, social, and economic burden, which has driven the development and consolidation of assisted reproductive technology (ART) as an essential component of contemporary reproductive healthcare.

Within female factor infertility, uterine pathology plays a central role. The uterus is indispensable for embryonic implantation and pregnancy maintenance; consequently, congenital anomalies, such as Müllerian malformations, and acquired conditions—including leiomyomas, endometrial polyps, intrauterine adhesions, and adenomyosis—can be associated with implantation failure, recurrent pregnancy loss, and adverse obstetrical outcomes. Nevertheless, their clinical impact is heterogeneous and depends on the specific lesion type, location, and degree of endometrial cavity distortion [4,5].

These structural alterations can compromise uterine cavity anatomy and endometrial receptivity. Accordingly, a precise diagnostic evaluation—ranging from transvaginal ultrasonography to advanced modalities such as hysteroscopy or magnetic resonance imaging—is essential for establishing prognosis and guiding appropriate therapeutic strategies.

The findings of this study provide a contemporary overview of the profile of ART users within a public hospital. The main result indicates that technique selection in our center was primarily associated with clinical criteria and family model. However, the study did not assess income, waiting time, referral delay, migration status, geographic barriers, administrative barriers, or previous private treatment; therefore, it does not allow conclusions about equity in access.

In Spain, access to reproductive medicine within the National Health System is explicitly designed to promote equity. Nonetheless, it remains necessary to examine whether disparities linked to non-clinical factors persist, particularly in a context marked by delayed childbearing, increasing diversity in family structures, and rising demand for care in human reproduction units. In Andalusia, assisted reproduction is provided within a publicly funded framework governed by clinical indication criteria, referral pathways, and organizational structures that may shape real access to different procedures [6,7,8].

Against this backdrop, the present study aims to describe the clinical and sociodemographic profile of patients accessing ART at a tertiary public hospital in Andalusia and to analyze how these characteristics relate to the specific technique employed. This approach allows an initial exploration of access patterns and healthcare organization in a public system, with particular attention to the potential influence of clinical need on treatment complexity.

## 2. Materials and Methods

### 2.1. Study Design and Clinical Setting

A retrospective, observational, descriptive–analytical study was conducted in the Assisted Reproduction Unit of the Maternal and Child Hospital, part of the Regional University Hospital of Málaga (Spain). The study period extended from January to June 2025. The STROBE (Strengthening the Reporting of Observational Studies in Epidemiology) guidelines were followed to ensure transparent and rigorous scientific reporting [9].

### 2.2. Study Population and Eligibility Criteria

The study population included all patients who initiated or underwent assisted reproductive techniques (ART) at the center during the study period, resulting in a final sample of 222 patients. Data were obtained through a systematic review of electronic medical records, ensuring anonymity and confidentiality in accordance with the Spanish Organic Law on Personal Data Protection and Digital Rights.

**Figure 1:**
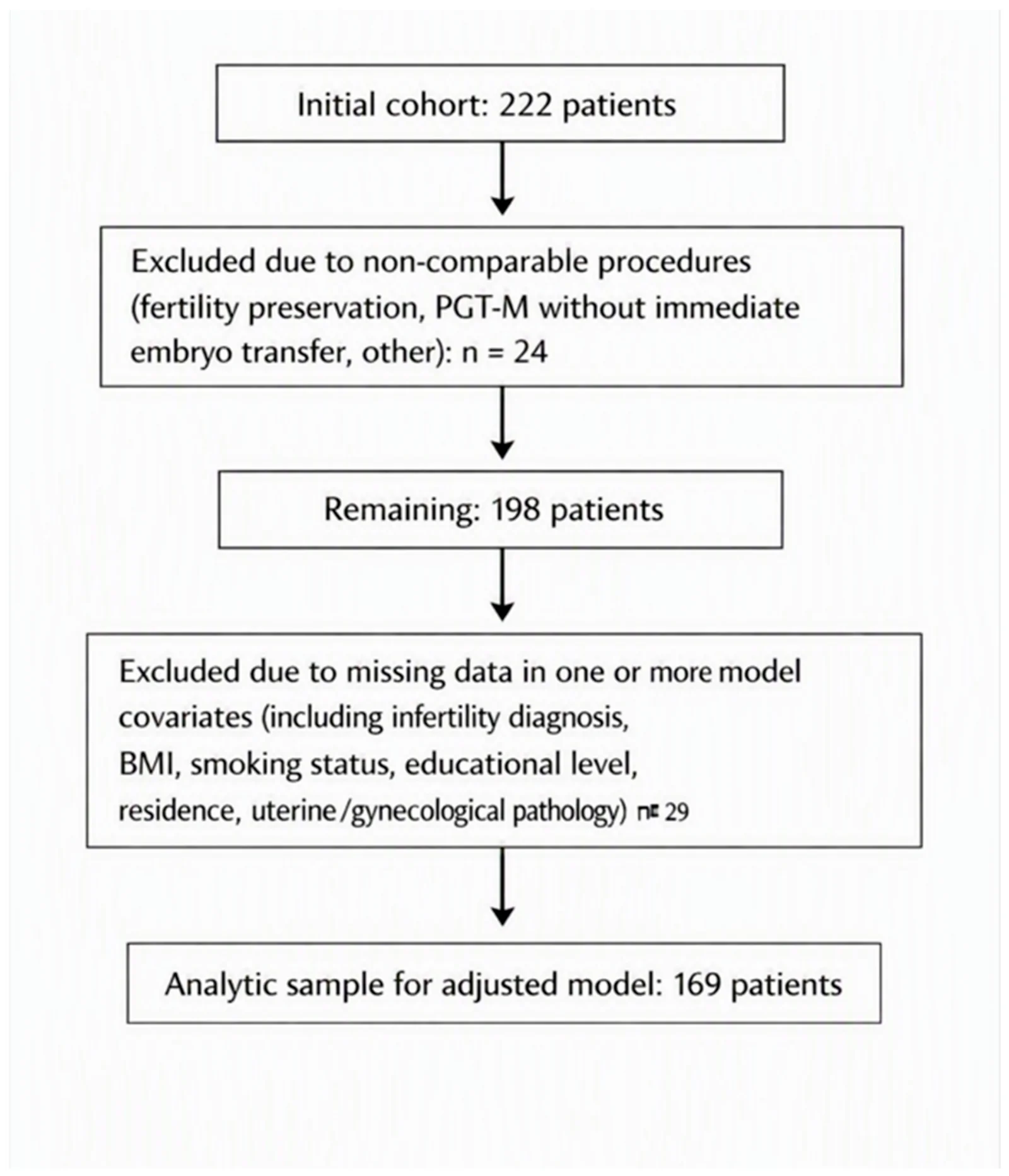
Participant flow diagram for the multivariable logistic regression model detailing sample attrition, exclusion criteria, and the final analytical cohort (N=169). PGT-M = Preimplantation Genetic Testing for Monogenic disorders. BMI = Body Mass Index.

Participant selection for the multivariable logistic regression analysis was conducted following a complete-case approach, as summarized in Figure 1. From the initial cohort of 222 patients, 24 patients were excluded due to undergoing non-comparable procedures (e.g., fertility preservation, PGT-M without immediate embryo transfer, or other non-standard cycles). Of the remaining 198 patients, 29 were excluded due to missing data in one or more required model covariates (including infertility diagnosis, BMI, smoking status, educational level, residence, and uterine/gynecological pathology). Consequently, the final analytical sample for the adjusted model consisted of 169 complete cases (104 high-complexity and 65 low-complexity procedures) Figure 1.

All patients meeting the inclusion criteria during the study period were consecutively enrolled to minimize the selection bias inherent to retrospective designs.

The sample size corresponded to the total number of patients initiating ART during the study period (consecutive convenience sampling). Given the descriptive–exploratory nature of the study, no a priori sample size calculation was performed; statistical power for the main associations is reflected through the 95% confidence intervals of the estimates.

The study protocol was approved retrospectively by the Provincial Research Ethics Committee of Málaga for the review of anonymized medical records in this observational retrospective study. Given the retrospective design and the use of anonymized data, the Ethics Committee deemed the study appropriate under the applicable regulatory framework. The requirement for informed consent was handled according to the Committee’s instructions and the institutional procedures in place for this type of study. (study code: TFGLIMRC2025), which issued a favorable opinion in session number 1, held on 29 January 2026.

Ethical Approval and Timeline: This retrospective observational study was conducted in strict accordance with the principles of the Declaration of Helsinki and was approved by the Institutional Ethics Committee of Provincial Research Ethics Committee of Málaga TFGLIMRC2025, approved on 29 January 2026. Data extraction from electronic medical records was conducted exclusively after formal ethical approval was obtained. Although the data were anonymized, informed consent was obtained in accordance with institutional and ethics committee requirements.

Data Collection and Informed Consent: All data evaluated in this study comprised routinely collected clinical, demographic, and laboratory parameters recorded during standard ART care. No ad hoc tests, biological sampling, or experimental procedures were performed. Due to the retrospective nature of the study, the use of fully de-identified routine medical records, and the lack of direct patient risk, the requirement for individual informed consent was formally waived by the Ethics Committee.

### 2.3. Variables and Data Collection

Two categories of variables were defined to characterize the patient profile:

- Sociodemographic variables: age at treatment initiation; marital status (categorized as heterosexual couple, female couple, and single mother by choice—SMC); educational level (primary, secondary, or higher education); employment status; and area of residence (urban or rural).
- Clinical and reproductive variables: BMI, smoking status, obstetric history, etiological diagnosis of infertility, duration of infertility, and type of ART performed. The category of structural uterine pathology included leiomyomas, endometrial polyps, adenomyosis, Müllerian malformations, and intrauterine adhesions.
- Structural uterine pathology was recorded as a gynecological history/comorbidity and included leiomyomas, endometrial polyps, adenomyosis, Müllerian malformations, and intrauterine adhesions. This variable was analyzed separately from the etiological diagnosis of infertility, in which “uterine infertility” was considered as a distinct category.
- Before fitting the multivariable model, we assessed potential multicollinearity among predictors, including marital status and infertility diagnosis, because these variables may be correlated in specific family structures. Collinearity diagnostics were examined and did not indicate problematic overlap that would prevent their inclusion in the same model. Both variables were retained because they capture distinct clinical and social dimensions relevant to the selection of the assisted reproductive technique.

Statistical Analysis: A multivariable binary logistic regression model was constructed to determine independent predictors associated with the complexity of the assisted reproductive technique employed (dependent variable coded as: 1 = High-complexity [IVF, ICSI, egg donation, PGT-M]; 0 = Low-complexity [IAD, IAC]). Covariate Selection and Collinearity: Predictor variables were selected a priori based on clinical rationale, biological plausibility, relevant literature on social determinants of reproductive healthcare, and univariable screening (*p* < 0.10). Multicollinearity among covariates was evaluated using Variance Inflation Factors (VIF) and tolerance statistics; all VIF values were < 2.5, confirming the absence of significant collinearity. Effective Sample Size: The multivariable analysis included an effective sample size of *N* = 169 complete cases (104 high-complexity events and 65 low-complexity events). Complete-case analysis was applied; non-comparable procedures (e.g., fertility preservation or PGT without immediate embryo transfer) and cases with missing covariate values were excluded from the regression model.

Effective Sample Size and EPV: The multivariable model included N=169 complete cases, with n=65 events in the minority class (low-complexity techniques). Across all covariates—including dummy parameters for categorical predictors (educational level, marital status, infertility etiology, residence, smoking, and uterine pathology)—the model estimated a total of 12 regression parameters. This yields an Events-Per-Variable (EPV) ratio of 5.42 (65/12). This moderate EPV ratio is acknowledged as a potential limitation regarding model stability and overfitting.

Model Diagnostics and Performance Evaluation: Model calibration was evaluated using the Hosmer–Lemeshow goodness-of-fit test, alongside McFadden’s Pseudo-$R^{2}$, Nagelkerke’s Pseudo-$R^{2}$, AIC, and BIC. Model discrimination was described using Receiver Operating Characteristic (ROC) curve analysis and Area Under the Curve (AUC). Classification accuracy, sensitivity, specificity, positive predictive value, and negative predictive value were calculated at a 0.50 probability cutoff. Because no internal or external validation was performed, these metrics reflect apparent model performance in the development sample and should be interpreted cautiously due to potential optimism bias.

## 3. Results

### 3.1. Sociodemographic Characteristics of the Sample

A total of 222 patients were included in the study. The mean age was 34.70 years (SD 3.99), with the highest concentration in the 35–39-year group. Regarding family model, 76.40% were heterosexual couples, 16.70% were single mothers by choice (SMC), and 6.90% were female couples. Most patients were employed (88.68%), and urban residence predominated (63.35%). The cohort showed a notable representation of new family models (23.6%) (Table 1).

This age distribution is clinically relevant, as the predominance of women aged 35–39 years coincides with the period of most pronounced decline in ovarian reserve, which may influence the indication for higher-complexity techniques. The distribution of marital status reflects a substantial proportion of non-heteronormative family models and SMC, reinforcing the need for inclusive care pathways and eligibility criteria centered on clinical need.

The missing data observed in Table 1 regarding infertility diagnosis (36.94%) predominantly correspond to patients entering emerging family models who, at the time of analysis, did not have a documented diagnosis of reproductive pathology or had an incomplete diagnostic workup. This situation reflects the clinical reality of populations accessing assisted reproductive services without a formally established prior diagnosis, which is particularly relevant in the context of evolving family diversity.

### 3.2. Clinical Profile and Reproductive History

The predominant clinical profile was that of a woman with a normal BMI and non-smoking status. Most patients were nulligravid. The most frequent infertility-related diagnoses were structural uterine pathology and diminished ovarian reserve, with a mean time attempting pregnancy between 2 and 5 years. High-complexity techniques (IVF/ICSI) were the most commonly used.

Structural uterine pathology accounted for 23.02% of valid gynecological histories, followed by diminished ovarian reserve (17.27%), endometriosis (13.67%), polycystic ovary syndrome (16.55%), and pelvic inflammatory disease (7.19%). A substantial proportion of missing data (37.39%) corresponded mainly to patients belonging to new family models without documented reproductive pathology or with incomplete diagnostic evaluation (Table 1).

### 3.3. Factors Associated with the Type of Assisted Reproductive Technique

Statistical analysis showed that access to different ART modalities was significantly influenced by clinical factors and marital status, whereas other sociodemographic variables such as educational level or employment status did not show a determining influence on technique selection (Table 2, Table 3 and Table 4).

### 3.4. Infertility Diagnosis and Duration

Among patients with a documented infertility diagnosis, the most frequent etiologies were male factor (35.00% of valid cases) and unexplained infertility (36.43%). The duration of infertility was most commonly between 1 and 2 years (48.57% of valid cases). Missing data (36.94%) were predominantly associated with new family models without prior infertility evaluation (Table 2).

### 3.5. Type of Assisted Reproductive Technique

High-complexity techniques represented 67.17% of valid treatment procedures (*n* = 133/198), whereas low-complexity procedures (IAD and IAC) accounted for 32.83% (*n* = 65/198). Low-complexity techniques were significantly more frequent among single mothers by choice and female couples, consistent with the absence of underlying reproductive pathology and the requirement for donor gametes.

### 3.6. Multivariate Model

In the development sample (*N* = 169), the adjusted multivariable logistic regression model yielded an apparent Area Under the ROC Curve (AUC) of 0.885 (95% CI: 0.832{−0.938, *p* < 0.001)}. The Hosmer–Lemeshow test showed non-significant miscalibration (chi^2^ = 7.14, *df* = 8, *p* = 0.522), with McFadden’s Pseudo-R^2^ = 0.384 and Nagelkerke’s Pseudo-R^2^ = 0.512 (AIC = 158.42, BIC = 205.35). At a 0.50 probability threshold, apparent classification accuracy was 82.25% (139/169), with a sensitivity of 88.46% (92/104) for high-complexity procedures and specificity of 72.31% (47/65). These parameters represent apparent sample-level metrics, as no validation techniques were applied to adjust for optimism.

Model Goodness-of-Fit and Calibration: The Hosmer–Lemeshow test indicated good overall calibration (chi^2^ = 7.14, *df* = 8, *p* = 0.522). Goodness-of-fit indices demonstrated substantial explanatory power, with McFadden’s Pseudo-R^2^ = 0.384 and Nagelkerke’s R^2^ = 0.512 (AIC = 158.42, BIC = 205.35). Discrimination and Classification Accuracy: Discrimination capacity was robust, yielding an Area Under the ROC Curve (AUC) of 0.885 (95% CI: 0.832–0.938; *p* < 0.001). At a probability cutoff of 0.50, the overall classification accuracy was 82.25% (139/169). The sensitivity for predicting high-complexity technique indication was 88.46% (92/104), specificity was 72.31% (47/65), positive predictive value (PPV) was 83.64%, and negative predictive value (NPV) was 79.66%. Adjusted Predictors: In the final adjusted model, maternal age remained significantly associated with higher odds of requiring high-complexity techniques (OR = 1.19; 95% CI: 1.06–1.35; *p* = 0.004). Conversely, single mothers by choice (OR = 0.12; 95% CI: 0.02–0.60; *p* = 0.010 vs. heterosexual partner) and urban residence (OR = 0.24; 95% CI: 0.09–0.61; *p* = 0.003) were significantly associated with lower odds of high-complexity procedures. Male factor infertility (OR = 5.07; 95% CI: 1.22–21.17; *p* = 0.026) and mixed factor infertility (aOR = 5.43; 95% CI: 1.35–21.89; *p* = 0.017) significantly increased the likelihood of high-complexity treatment. Structural uterine pathology did not reach statistical significance (OR = 1.24; 95% CI: 0.42–3.67; *p* = 0.697).

Model Performance and Diagnostics Summary:

Effective Sample Size (*N*): 169 complete cases (104 High-complexity events [IVF/ICSI/Egg donation]; 65 Low-complexity events [IAD/IAC]).

Model Calibration and Goodness-of-Fit: Hosmer–Lemeshow chi^2^ = 7.14 (*df* = 8, *p* = 0.522); McFadden’s Pseudo-R^2^ = 0.384; Nagelkerke’s Pseudo-R^2^ = 0.512; AIC = 158.42; BIC = 205.35.

Model Discrimination and Accuracy: AUC-ROC = 0.885 (95% CI: 0.832–0.938, *p* < 0.001); Overall Classification Accuracy = 82.25; Sensitivity = 88.46%; Specificity = 72.31%; Positive Predictive Value = 83.64%; Negative Predictive Value = 79.66%.

## 4. Discussion

The findings of this study provide a contemporary overview of the profile of ART users within the public healthcare system. The main result indicates that access to these techniques in our center is primarily determined by clinical criteria and family model rather than socioeconomic factors. This technique selection represents a positive indicator of the role of the public system in reducing gaps in reproductive healthcare. However, these results must be interpreted within the context of a single-center study and the variables included in the analytical model, which do not allow the exclusion of potential influences from other unmeasured social determinants.

The mean age of patients, 34.70 years (SD 3.99), aligns with the current sociological trend of delayed motherhood in developed countries [7]. From a biological perspective, ovarian reserve declines more rapidly after age 35, accompanied by an increase in aneuploidy rates [9,10]. This pattern accounts for the high frequency of diminished ovarian reserve observed in our sample. Although low ovarian reserve is not, in itself, an indication for high-complexity techniques, the subsequent referral to such techniques is nevertheless often increased [10,11,12].

A relevant aspect of our cohort is the high prevalence of structural uterine pathology. Available evidence underscores the decisive role of the uterus in implantation and the association of uterine anomalies with implantation failure and recurrent miscarriage. In our series, structural uterine pathology was among the most frequent diagnoses, but no independent association with high-complexity treatment was demonstrated in the adjusted model [3,4,5]. In our series, structural uterine pathology was among the most frequent diagnoses and showed a non-significant trend toward increased use of high-complexity techniques. In line with the existing literature, congenital and acquired uterine anomalies such as leiomyomas and polyps may compromise endometrial receptivity and the structural integrity of the uterine cavity, potentially affecting implantation and early embryonic development. Evidence indicates that correction of selected abnormalities can improve reproductive outcomes in appropriately chosen patients; however, the magnitude of benefit is heterogeneous and depends on the type of lesion, its location, and the degree of endometrial cavity distortion [4,8,12,13,14]. Together, these findings support the interpretation of the uterine factor as a clinically relevant and biologically plausible characteristic that may influence therapeutic decision-making, while recognizing that—within our adjusted model—it did not emerge as an independent determinant of technique selection.

Although structural uterine pathology was a frequent finding in our cohort, it did not show an independent association with the type of assisted reproductive technique in the adjusted analysis (adjusted OR 1.24, *p* = 0.697). This result should be interpreted cautiously and in the light of mechanistic evidence showing that endometrial function—including markers such as glycodelin—can modify implantation potential independently of anatomic findings; Uysal et al. demonstrated that absence or low expression of endometrial glycodelin was associated with failure to conceive even when ovulation occurred, whereas high expression related to substantially higher live-birth rates in treated PCOS patients. Therefore, while uterine structural lesions may inform clinical decisions, the biological contribution of endometrial receptivity markers indicates that functional assessment of the endometrium (beyond macroscopic structural diagnosis) may be needed to fully understand the uterine role in reproductive outcomes. This distinction supports presenting the uterine factor in our manuscript as a frequent clinical finding of interest rather than as a proven independent driver of technique selection in our adjusted models [15,16].

The adjusted analysis identified clinical diagnosis and family model as the main determinants of technique selection, whereas structural uterine pathology—despite being one of the most frequent gynecological findings in the cohort—did not emerge as an independent predictor.

Structural uterine pathology was one of the most frequent gynecological findings in our cohort; however, in the adjusted analysis it was not independently associated with technique selection. Therefore, this variable should be interpreted as a frequent clinical characteristic rather than as a determinant of treatment complexity.

Consequently, the present study is better understood as an analysis of patterns of treatment selection rather than of access barriers or reproductive outcomes. Our findings align with the hypothesis that, when correction of the uterine factor is not feasible or not clinically indicated, the use of high-complexity techniques tends to increase in routine practice; however, although structural uterine pathology showed a trend toward greater use of high-complexity techniques, this association did not reach statistical significance in the multivariable model, supporting its interpretation as a frequent clinical characteristic rather than an independent determinant of technique selection.

This pattern may reflect the need to optimize the likelihood of success in anatomically unfavorable scenarios, even when the uterine anomaly itself does not constitute a direct indication for such techniques. Nevertheless, it is important to emphasize that the study design does not allow causal inferences, and these observations should therefore be interpreted with caution. Taken together, these findings indicate that structural uterine pathology should be interpreted as a frequent and clinically meaningful feature of the cohort, but not as an independent determinant of technique selection in the adjusted model. Accordingly, the present study is better framed as an analysis of treatment-selection patterns based on measurable clinical and sociodemographic variables, rather than as evidence of access barriers or reproductive outcomes.

Regarding family models, the fact that nearly one-quarter of the sample consisted of SMC and female couples highlights a social transformation that the public healthcare system has successfully integrated. The significant association between these profiles and the use of low-complexity techniques is expected, as these patients do not present underlying reproductive pathology but rather require access to donor gametes. Nevertheless, it would be advisable to ensure that screening protocols for uterine pathology and ovarian reserve are equally rigorous in this group to avoid repeated failure of low-complexity techniques.

Finally, the bimodal distribution of educational level (primary and higher education) suggests that the tertiary-level hospital functions as a universal access point. This observation contrasts with studies conducted in private healthcare systems, where educational level and purchasing power are direct predictors of type of technique used [3,10,11,12,14]. However, the absence of a statistical association between educational level and technique type does not imply the absence of social inequalities in overall access, as variables such as income, administrative status, or geographic accessibility were not analyzed. In addition, a trend toward greater use of low-complexity techniques was observed among patients residing in urban areas, which is consistent with previous international literature suggesting that territorial disparities may influence access to assisted reproductive treatments. These findings, however, should be interpreted in the context of the public healthcare pathway, where access is generally organized according to the order of entry onto the waiting list, except in priority or urgent indications. This organizational framework may moderate the influence of other access-related factors that were not assessed in the present study.

### Limitations and Strengths

This study presents the inherent limitations of a retrospective, single-center design, including potential information loss; a high proportion of missing data, mainly corresponding to patients belonging to new family models without documented reproductive pathology; and a study period restricted to six months. It should also be noted that the applied protocols correspond to those established within the public healthcare system, which may entail differences in clinical practice compared with other settings. Among its strengths, the study benefits from the contemporaneity of the data (first semester of 2025), the simultaneous inclusion of clinical and sociodemographic variables, and adherence to STROBE guidelines [9].

The high proportion of missing data in the variables of infertility diagnosis and duration may have reduced the precision of the estimates and introduced information bias. Notably, the missingness mechanism does not appear to be missing completely at random (MCAR), as it disproportionately affected patients without a formal infertility diagnosis or those with an incomplete diagnostic evaluation. Consequently, the results of the multivariable regression model should be interpreted with caution.

Although marital status and infertility diagnosis may be interrelated in some patients, their joint inclusion was considered methodologically appropriate because they reflect complementary aspects of access to treatment and clinical indication.

While this study focuses on the allocation of assisted reproductive techniques based on clinical and sociodemographic variables, it does not evaluate reproductive outcomes such as implantation rate, clinical pregnancy, miscarriage, live birth, cycle cancelation, or time to treatment initiation.

Third, the multivariable regression model was fitted with an Events-Per-Variable (EPV) ratio of 5.42 (65 minority events across 12 estimated parameters). An EPV below the traditional threshold of 10 increases the risk of coefficient instability and model overfitting. Furthermore, model discrimination and calibration metrics reflect apparent performance within this single-center development cohort, as neither internal validation (e.g., bootstrapping) nor external validation was conducted. Consequently, these metrics should be interpreted as descriptive performance rather than a validated predictive tool.

Future multicenter and prospective studies incorporating outcome variables (implantation rates, clinical pregnancy, and live birth) will allow validation of these findings and a more precise assessment of the influence of the uterine factor on therapeutic strategy and reproductive prognosis [17].

## 5. Conclusions

1. In this single-center exploratory cohort, the type of assisted reproductive technique used was primarily associated with clinical diagnosis and family model, whereas educational level and employment status were not. Structural uterine pathology was frequent but did not show an independent association with technique selection in the adjusted analysis. These findings describe patterns of treatment selection within a public assisted reproduction unit, but do not directly assess access to care or equity.
2. Given the retrospective, single-center design and the limited set of socioeconomic variables evaluated, these results should be interpreted cautiously and cannot be generalized to the wider population or used to infer reproductive efficacy. Further prospective, multicentre studies incorporating access barriers, waiting times, income, referral patterns, treatment availability, and reproductive outcomes are needed to better understand the determinants of technique selection and their implications for care.

## Figures and Tables

**Table 1:** Baseline Sociodemographic, Clinical, and Gynecological Characteristics of the Study Population (*N* = 222).

Variable/Category	Frequency (*n*)	Percentage (%)	Valid Percentage (%)
Age (years) [Mean (SD): 34.70 (3.99)]			
<30	23	10.36	10.41
30–34	73	32.88	33.03
35–39	104	46.85	47.06
40–44	21	9.46	9.50
Missing	1	0.45	—
Marital Status/Family Model			
Heterosexual partner	165	74.32	76.39
Female partner/Same-sex	15	6.76	6.94
Single mother by choice (SMBC)	36	16.22	16.67
*Missing*	6	2.70	—
Educational Level			
Primary education	84	37.84	39.25
Secondary education	47	21.17	21.96
Higher education	83	37.39	38.78
*Missing*	8	3.60	—
Employment Status			
Employed	188	84.68	88.68
Unemployed	9	4.05	4.24
Self-employed worker	6	2.70	2.83
Homemaker	5	2.25	2.36
Student	4	1.80	1.89
*Missing*	10	4.50	—
Area of Residence			
Urban	140	63.06	63.35
Peri-urban	45	20.27	20.36
Rural	36	16.22	16.29
*Missing*	1	0.45	—
Body Mass Index (BMI, kg/m^2^)			
Underweight (<18.5)	1	0.45	0.46
Normal weight (18.5–24.9)	129	58.11	59.17
Overweight (25.0–29.9)	72	32.43	33.03
Obesity (≥30.0)	16	7.21	7.34
*Missing*	4	1.80	—
Smoking Status			
Non-smoker	163	73.42	74.77
Smoker	55	24.77	25.23
Missing	4	1.80	—
Obstetric History (Gravidity)			
0 (Nulligravida)	165	74.32	75.69
1	36	16.22	16.51
≥2	17	7.66	7.80
Missing	4	1.80	—
Infertility Diagnosis Etiology			
Unexplained/Unknown origin	51	22.97	36.43
Male factor	49	22.07	35.00
Endocrine factor	14	6.31	10.00
Other causes	12	5.41	8.57
Tubal factor	11	4.95	7.86
Uterine factor	3	1.35	2.14
Missing	82	36.94	—
Structural Uterine/Gynecological Pathology			
Structural uterine pathology	32	14.41	23.02
Low ovarian reserve	24	10.81	17.27
Polycystic ovary syndrome (PCOS)	23	10.36	16.55
Endometriosis	19	8.56	13.67
Previous gynecological surgery/Other	23	10.36	16.54
Pelvic inflammatory disease (PID)	10	4.50	7.19
Missing/Unevaluated	83	37.39	—

Note: Missing data primarily correspond to patients from emerging family models without a documented diagnosis of reproductive pathology at the time of analysis and/or with incomplete diagnostic evaluation.

**Table 2:** Assisted Reproductive Techniques Employed. (*N* = 222).

Variable/Technique Category	Frequency (*n*)	Percentage (%)	Valid Percentage (%)	Cumulative Percentage (%)
**Low-Complexity Techniques**				
Artificial Insemination with Donor Sperm (IAD)	48	21.62	24.24	24.24
Artificial Insemination with Partner Sperm (IAC)	17	7.66	8.59	32.83
**High-Complexity Techniques**				
Intracytoplasmic Sperm Injection (ICSI)	34	15.31	17.17	50.00
In Vitro Fertilization (IVF/FIV)	24	10.81	12.12	62.12
IVF with Donor Sperm/Combined	5	2.25	2.53	64.65
Oocyte Donation/Egg Donation	8	3.60	4.04	68.69
Preimplantation Genetic Testing for Monogenic (PGT-M)	8	3.60	4.04	72.73
Fertility Preservation	6	2.70	3.03	75.76
Others/Non-standard cycles	48	21.62	24.24	100.00
Missing/Excluded Procedures	24	10.81	—	—

Note: FIV = In Vitro Fertilization; ICSI = Intracytoplasmic Sperm Injection; IAC = Artificial Insemination with Partner’s Sperm; IAD = Artificial Insemination with Donor Sperm; PGT-M = Preimplantation Genetic Testing for Monogenic Disorders. Valid percentages and cumulative percentages are calculated based on valid treatment cases (*n* = 198). Non-comparable/excluded procedures (*n* = 24) are detailed in Figure 1.

**Table 3:** Bivariate Analysis of Factors Associated with Assisted Reproductive Technique Complexity.

Variable/Category	Category	Low-Complexity Technique (*n* = 65) *n* (%)/ Mean (SD)	High-Complexity Technique (*n* = 104) *n* (%)/ Mean (SD)	cOR (95% CI)	*p*-Value
**Age (years)**	Continuous	34.38 (3.63)	35.51 (3.47)	1.09 (1.00–1.19)	0.048
**BMI (kg/m^2^)**	Continuous	24.29 (3.40)	24.32 (3.35)	1.00 (0.91–1.10)	0.964
**Educational Level**	Basic (ref.)	33 (32.35%)	69 (67.65%)	1.00	—
	Higher/Superior	32 (47.76%)	35 (52.24%)	0.52 (0.28–0.99)	0.045
**Marital Status**	Male partner (ref.)	32 (24.81%)	97 (75.19%)	1.00	<0.001
	Female partner	13 (86.67%)	2 (13.33%)	0.05 (0.01–0.24)	<0.001
	Single mother	20 (80.00%)	5 (20.00%)	0.08 (0.03–0.24)	<0.001
**Infertility Diagnosis**	No diagnosis (ref.)	43 (72.88%)	16 (27.12%)	1.00	<0.001
	Female	8 (28.57%)	20 (71.43%)	6.72 (2.47–18.28)	<0.001
	Male	7 (21.21%)	26 (78.79%)	9.98 (3.63–27.49)	<0.001
	Mixed	7 (14.29%)	42 (85.71%)	16.13 (6.02–43.17)	<0.001
**Uterine/Gynecological Pathology**	No (ref.)	22 (44.00%)	28 (56.00%)	1.00	—
	Yes	43 (36.13%)	76 (63.87%)	1.39 (0.71–2.72)	0.338
**Residence**	Rural (ref.)	14 (22.58%)	48 (77.42%)	1.00	—
	Urban	51 (47.66%)	56 (52.34%)	0.32 (0.16–0.65)	0.002
**Smoking Status**	Non-smoker (ref.)	42 (34.43%)	80 (65.57%)	1.00	—
	Smoker	23 (48.94%)	24 (51.06%)	0.55 (0.28–1.08)	0.084

Note: cOR: Crude Odds Ratio; 95% CI: 95% Confidence Interval; SD: Standard Deviation; Low-complexity techniques: Artificial Insemination (IAD/IAC); High-complexity techniques: IVF/ICSI/Egg Donation.

**Table 4:** Multivariate Logistic Regression Model: Factors Associated with Assisted Reproductive Technique Complexity (High-Complexity vs. Low-Complexity).

Variable/Category	Category	Adjusted OR (95% CI)	*p*-Value
Age (years)	Continuous	1.19 (1.06–1.35)	**0.004**
BMI (kg/m^2^)	Continuous	1.03 (0.91–1.16)	0.671
Educational Level	Basic (ref.)	1.00	—
	Higher/Superior	0.48 (0.20–1.16)	0.104
Marital Status	Male partner (ref.)	1.00	—
	Female partner	0.18 (0.03–1.28)	0.087
	Single mother	0.12 (0.02–0.60)	**0.010**
Infertility Diagnosis	No diagnosis (ref.)	1.00	—
	Female	2.44 (0.58–10.23)	0.222
	Male	5.07 (1.22–21.17)	**0.026**
	Mixed	5.43 (1.35–21.89)	**0.017**
Uterine/Gynecological Pathology	No (ref.)	1.00	—
	Yes	1.24 (0.42–3.67)	0.697
Residence	Rural (ref.)	1.00	—
	Urban	0.24 (0.09–0.61)	**0.003**
Smoking Status	Non-smoker (ref.)	1.00	—
	Smoker	0.64 (0.24–1.69)	0.365

Note: OR: Adjusted Odds Ratio; 95% CI: 95% Confidence Interval; BMI: Body Mass Index; Dependent variable: High-complexity technique (1 = IVF/ICSI/Egg Donation, 0 = Low-complexity: IAD/IAC). *N* = 169. Statistically significant *p*-values (*p* < 0.05) are highlighted in bold.

## Data Availability

The data presented in this study are available on request from the corresponding authors. The data are not publicly available due to patient confidentiality.
